# Root vertical distributions of two *Artemisia* species and their relationships with soil resources in the Hunshandake desert, China

**DOI:** 10.1002/ece3.6135

**Published:** 2020-03-04

**Authors:** Xiuli Gao, Xiaoqiang Liu, Linna Ma, Renzhong Wang

**Affiliations:** ^1^ State Key Laboratory of Vegetation and Environmental Change Institute of Botany The Chinese Academy of Sciences Beijing China; ^2^ University of Chinese Academy of Sciences Beijing China

**Keywords:** *Artemisia* species, root traits, soil moisture and nutrients, the Hunshandake desert

## Abstract

1. Plant root variations and their relations with soil moisture and nutrient supply have been well documented for many species, while effects of drought, combined with extreme poor soil nutrients, on plant roots remain unclear.
2. Herein, we addressed root vertical distributions of two typical xerophyte semishrub species, *Artemisia sphaerocephala* and *A. intramongolica*, and their relations with soil moisture, total soil nitrogen and carbon contents in arid Hunshandake desert, China. The two species experienced similar light regimes and precipitation, but differed in soil moisture and soil nutrients.
3. Root vertical distribution patterns (e.g., coarse root diameter, root depth and root biomass) differed considerable for the two species due to high heterogeneity of soil environments. Coarse and fine root biomasses for *A. intramongolica*, distributed in relatively moist fixed dunes, mainly focused on surface layers (94%); but those for *A. sphaerocephala* dropped gradually from the surface to 140 cm depth. Relations between root traits (e.g., diameter, root biomass) and soil moisture were positive for *A. intramongolica*, but those for *A. sphaerocephala* were negative.
4. In general, the root traits for both species positively correlated with total soil nitrogen and carbon contents. These findings suggest that both soil moisture and poor soil nutrients were the limiting resources for growth and settlement of these two species.

## Graphical abstract

The root traits for *Artemisia sphaerocephala* and *A. intramongolica* were positively correlated with total soil nitrogen and carbon contents. These findings indicate that both soil moisture and poor soil nutrients were the limiting resources for growth in these two species.

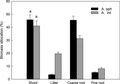

## 1 INTRODUCTION

Roots not only fix plants to soil, but also an important cycling interface of water and nutrients in terrestrial ecosystems, and relationships of root traits with soil water and nutrients can well reflect the specific adaptation and species ability to survive and settle in harsh environments (e.g., saline lands and deserts). In general, the availability of water and soil nutrients to plants in an ecosystem, where annual evaporation demand exceeds precipitation, depends on local climate, edaphic factors and on the depth, lateral spread and degree of overlap of plant root systems (Balogianni, Wilson, Vaness, MacDougall, & Pinno, 2014; Casper & Jackson, 1997; Henry, Cal, Batoto, Torres, & Serraj, 2012; Nadelhoffer & Raich, 1992; Schenk & Jackson, 2002; Schulze et al., 1996). Shallow root systems are the attributes of some desert plants, but desert and temperate coniferous forests have the deepest rooting profiles of terrestrial biomes (Canadell et al., 1996; Hasse et al., 1996). Plants are typically predicted to have large root‐to‐shoot ratios and well‐developed root systems in dry conditions (Canadell et al., 1996). Water and nutrients uptake are maximized by increasing root investment, whereas the high biomass allocation to roots may reduce primary production because of the increase in maintenance respiration of roots (Wang & Gao, 2003). Moreover, some studies have proven that low levels of soil water and nutrients can promote preferential production of root biomass in dry environments (Archer, Quinton, & Hess, 2002; Bai, Wang, Chen, Zhang, & Li, 2008) and hypothesis that plants preferentially allocate more resources to roots when water and nutrients become limited to growth (Garnier, 1991). These works provide strong evidence that root production correlates with climate (e.g., precipitation, aridity), soil texture, and nutrient contents (McCormack et al., 2015; Wang & Gao, 2003), but previous research in water‐limited environments leaves little doubt about the relationships of plant roots with extreme poor soil nutrients combined with drought. This information is essential for formulating generalizations regarding the relationships of root traits with soil water and nutrients in dryland ecosystems.

*Artemisia sphaerocephala* Krasch and *A. intramongolica* H. C. F. are two dominant semishrubs in large areas of mobile dunes and low fixed dunes in the deserts of North China. Xerophytic traits, for example, relative deep roots and mucilaginous achene (Huang, Yitzchak, Hu, & Zhang, 2001), enable these species to successfully tolerate drought, well growth and settle when surface soil moisture is less than 2% in dry seasons. These two species were introduced not only for desertification land restoration in the Hunshandake desert since the early 1990s, but also for winter pastures in some regions. Previous studies on the two species have mainly focused on geographical distributions (Ma, 1993; Yang, Huang, Zhang, & Cornelissen, 2015), morphology and structure of mucilaginous achene and seed germination (Huang et al., 2001). However, the root traits of the two species and their relationships with low soil moisture and poor nutrients remain unclear in water‐limited, sandy environments. The aims of the present study were to investigate the root vertical distributions for the two *Artemisia* species, and to analyze their relations with soil moisture, low total soil nitrogen, and carbon contents. This knowledge is important for better understanding of plant underground biological processes in the habitats of extreme poor soil nutrients, combined with drought, in water‐limited regions. Furthermore, our specific aim was to provide reasonable basis for species selection in desertification land restoration in the Hunshandake desert, by compare root traits (*e.g*., root vertical distribution and biomass) of the two species and their relations with soil moisture and nutrients.

## 2 MATERIALS AND METHODS

### 2.1 Study site

The Hunshandake desert covers an area of about 31,500 km^2^ in the south Xilingol steppe, in the middle of Mongolian Plateau, North China. Sixty percent of the desert is fixed dunes and 20% is mobile dunes. The landscape comprises large areas of sand‐covered land mixed with *Stipa* (*Stipa krylovii*) steppes, meadows (dominated by *Leymus chinensis*), and a few farmlands, and the average altitude of the area is 1,200 m above sea level, varying from 1,050 m to 1,350 m. Because of human activities (e.g., overgrazing, cultivation and road construction), desertification is becoming serious in the last two decades and the region has become one of the sand storm sources in North China. More than 80% of the Hunshandake desert (42°07′~43°52′N, 111°35′~117°44′E) has sandy soil with gravel, while light chestnut occurs on steppes, meadows, and farmlands. Most of the desert is dominated by xerophytes, for example, *A. sphaerocephala* Krasch., *A. intramongolica* H. C. Fu. *S. grandis* P. Smirn., *S. gobica* Roshev. and *S. krylovii* Roshev. *A. sphaerocephala* and *A. intramongolica* were mixed sowing for desertification land restoration in the area in 2000. The two species form large patches of consociation, respectively, after two decades. With the vegetation dynamic, *A. sphaerocephala* distributed on the tops of mobile dunes (ranging 20 m to 100 m above the nearby steppes), while *A. intramongolica* was mainly on the low fixed dunes with 1–3 cm layer litter cover.

### 2.2 Climate

The Hunshandake desert is near the center of the Asian continent, which leads to a continental climate and low precipitation. In winter, the climate of the area is dominated by the intense Mongolian anticyclone, which produces a strong westerly flow of cold, dry continental air and little snowfall. As the anticyclone breaks down in spring, the region comes increasingly under the influence of moist Pacific air masses, reaching a climax in the summer monsoon which lasts two months. As the summer draw to an end, the low‐pressure area over the Indo‐Pakistan subcontinent disappears with the development of the Mongolian anticyclone. The mean annual air temperature ranges from −0.2°C to 2.0°C, varying from −21.6°C in January to 19.6°C in July. Annual precipitation varies from 100 mm in the west to 380 mm in the east. Precipitation is not distributed evenly over the growing season, of which 70% falls between June and August.

### 2.3 Methods

Root vertical distributions were investigated for *A. sphaerocephala* and *A. intramongolica* in Sandy Land Reservation Station in southern part of the Hunshandake desert. The two species were introduced for desertification land restoration in 2000, and each sample plant was over 15 years old. Three typical distribution sites (>1 ha large patch of consociation), at least 2 km apart, were chosen for each species, and more than 10 plants (excluding seedlings) were sampled randomly in each site. Forty‐five and forty‐one plants were sampled for *A. sphaerocephala* and *A. intramongolica*, respectively. The sample site for each species was of uniform soil type, with an even distribution of the *Artemisia* species (distribution frequency over 70%). Plants were sampled using 1 m × 1 m quadrat; then, the sandy soil was removed at 20 cm increments with spade carefully, until to the end of taproots (about 140 cm). Root vertical lengths and diameters for coarse roots (diameter > 2 mm) and fine roots (diameter < 2 mm) in each increment were measured with electronic vernier caliper (Acemeter Limited Company), and samples of each plant (coarse roots and fine roots) were placed in perforated paper bags separately and oven‐dried at 80°C to constant weight before weighing. Because of the plants were sampled in patches of consociation, there were no other plant roots should be removed. Biomass allocation referred to the biomass of root/shoot as a percent of total plant biomass.

About 150 g soil sample was taken in each increment of every sample quadrat. Meanwhile equivalent soil samples were taken in three adjacent *Stipa* steppe sites with light chestnut (at least 1.5 km apart from the *Artemisia* sample sites), in order to confirm that the soil condition was extreme poor nutrients, combined with drought, in the *Artemisia* sample sites by comparing with those of nearby Stipa steppes. Gravimetric soil moisture (SM) was measured by oven‐drying samples at 105°C for 24 hr, and the equation for calculating the SM = (Fresh weight–Dry weight)/Dry weight × 100%. Soil nitrogen and carbon contents were determined by using Kjeldahl method and potassium dichromate titration (Yuan, Ma, Guo, & Wang, 2016), respectively. Plant roots and soil samples were taken twice in early June (dry season) and later August (rainy season) for a comprehensive comparison of the differences between the two species in whole growing season. Although the seepage of sand soil was very well, plant roots and soil samples were taken a week after rain in rainy season in order to reducing the effects of rainfall.

### 2.4 Statistical analyses

The data of root lengths and biomass for each plant, taken from 20 cm increments, were combined as a whole. Mean coarse and fine root biomass and root lengths and diameters of the two species were statistically analyzed with SPSS 17.0 (SPSS for Windows). Significant differences between the two species in coarse and fine root biomass, lengths, and diameters were compared using one‐way analysis of variance (ANOVA). In order to explain the relationships between root parameters and soil attributes, regression of root parameters, for example, root biomass, diameters and lengths against soil moisture, nitrogen, and carbon contents were performed using SPSS 17.0.

## 3 RESULTS

### 3.1 Edaphic condition

Both soil moisture and nutrients from the *Artemisia* sample sites were significant lower than that from nearby steppe (Table 1). Average surface soil moisture (0–40 cm) in mobile dunes and fixed dunes was only 10%–40% of the adjacent *Stipa* steppe in the growing season due to low canopy cover and high evaporation. Total soil nitrogen and carbon contents for mobile dunes and fixed dunes were only 4%–18% of the steppe (Table 1) due to the output by sand storms. The *A. intramongolica* sites had relatively higher soil moisture, nitrogen, and carbon contents on surface layers (0–40 cm) than those from *A. sphaerocephala*.

**Table 1:** The average soil moisture (SM), total nitrogen (TN), and total carbon (TC) contents in the sites of *Artemisia sphaerocephala*, *A. intramongolica,* and adjacent *stipa* steppe in the Hunshandake desert

Deep layer (cm)		*Artemisia sphaerocephala*			*Artemisia intramongolica*			*Stipa* steppe		
		SM (%)	TN (%)	TC (g/kg)	SM (%)	TN (%)	TC (g/kg)	SM (%)	TN (%)	TC (g/kg)
I	0–20	0.85 ± 0.122	0.0056 ± 0.0007	0.46 ± 0.038	3.73 ± 0.411	0.0388 ± 0.0041	3.89 ± 0.288	10.16 ± 0.941	0.1901 ± 0.021	12.56 ± 1.986
II	21–40	1.33 ± 0.220	0.0061 ± 0.0004	0.32 ± 0.029	2.36 ± 0.392	0.0091 ± 0.0008	0.83 ± 0.076	10.77 ± 0.822	0.1013 ± 0.009	7.07 ± 0.975
III	41–60	1.34 ± 0.165	0.0052 ± 0.0004	0.27 ± 0.031	1.36 ± 0.220	0.0071 ± 0.0009	0.40 ± 0.039	11.55 ± 1.031	0.0659 ± 0.008	6.85 ± 0.789
IV	61–80	1.61 ± 0.249	0.0060 ± 0.0008	0.19 ± 0.021	1.49 ± 0.176	0.0052 ± 0.0007	0.38 ± 0.041	12.32 ± 0.988	0.0589 ± 0.007	6.07 ± 0.694
V	81–100	1.74 ± 0.120	0.0041 ± 0.0003	0.13 ± 0.014	2.04 ± 0.263	0.0048 ± 0.0006	0.31 ± 0.040	12.44 ± 1.326	0.0309 ± 0.005	3.86 ± 0.468
VI	101–120	2.25 ± 0.187	0.0040 ± 0.0003	0.10 ± 0.015	2.44 ± 0.189	0.0041 ± 0.0004	0.11 ± 0.019	14.29 ± 1.118	0.0286 ± 0.003	2.20 ± 0.321
VII	121–140	2.65 ± 0.166	0.0038 ± 0.0005	0.08 ± 0.009	2.72 ± 0.212	0.0037 ± 0.0005	0.09 ± 0.008	21.29 ± 1.967	0.0263 ± 0.003	2.18 ± 0.297

### 3.2 Root diameter and depth

**Figure 1:**
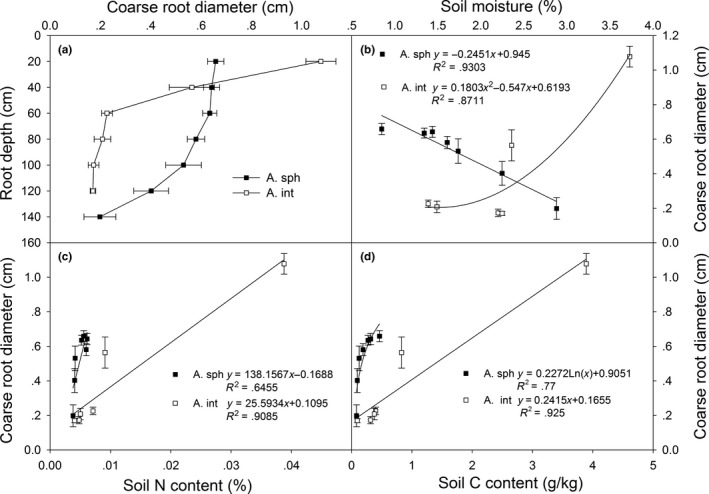
Coarse root diameters of *Artemisia sphaerocephala* (A. sph) and *A. intramongolica* (A. int) (a) and their relationships with soil moisture (b), total soil nitrogen (c), and total carbon content (d) in the Hunshandake desert, China

Coarse root diameter and root depth for the two *Artemisia* species varied in different patterns from the surface to 140 cm deep in Hunshandake desert (Figure 1a). Coarse root diameters for *A. sphaerocephala* dropped gradually with depth, and the differences between the layers from 61 cm to 140 cm were significant (*p* < .05). Average coarse root diameter in the surface layer (0–20 cm) was 64% greater than that in the VII layer (121–140 cm), and the latter was less than 2 mm. Root diameters for *A. intramongolica*, however, dropped sharply from the surface to the IV layer. The average coarse root diameter for the species in the surface layer was about 5 times of that in the latter layer, and the difference was significant (*p* < .001). Root diameter for the species was less than 2 mm in layers deeper than 80 cm, and the differences were not significant from 61 cm to 120 cm (*p* > .05).

Rooting depth for *A. sphaerocephala* was much greater than that for the *A. intramongolica* in the region (Figure 1a). Average coarse root depth for the former species was about 120 cm, while that for *A. intramongolica* was less than 80 cm. The fine roots for *A. intramongolica* were as deep as 120 cm, but those for *A. sphaerocephala* extended to 140 cm in deep. Significant differences in root diameter and root depth (*p* < .01) for the two *Artemisia* species may indicate their diverse growth patterns and inter‐specific differences.

Root diameters for *A. sphaerocephala* were negatively correlated with soil moisture from the surface layer to 140 cm deep (*p* < .01), while that for *A. intramongolica* were positively correlated with soil moisture but nonlinearly (*p* < .01, Figure 1b). Root diameters for both species were positively correlated with total soil nitrogen content (*p* < .05, Figure 1c) and soil carbon content (*p* < .01, Figure 1d).

### 3.3 Root biomass

**Figure 2:**
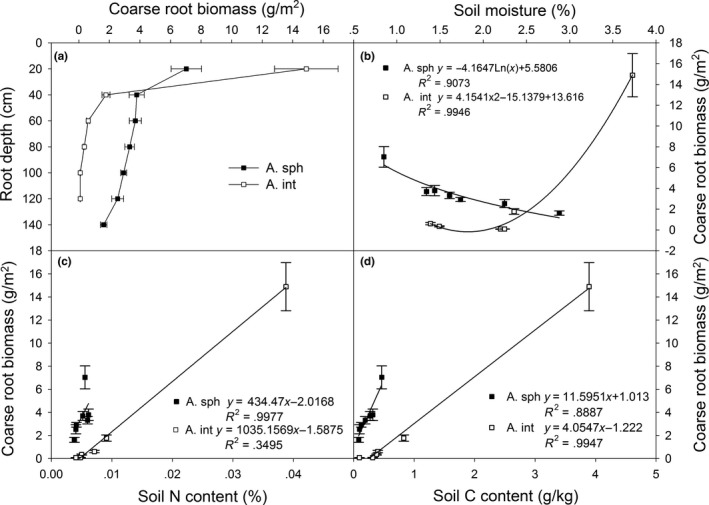
The coarse root biomsses for *Artemisia sphaerocephala* (A. sph) and *A. intramongolica* (A. int) (a) and their relationships with soil moisture (b), total soil nitrogen content (c), and total carbon content (d) in the Hunshandake desert, China

Coarse root biomass for the two species exhibited some different patterns with soil depth (Figure 2a). Ninety‐four percent of the coarse root biomass for *A. intramongolica* was in the first two layers, <1% of the coarse root biomass in the 81–120 cm layers. The majority (93%) of coarse root biomass for *A*. *sphaerocephala* was evenly distributed from the II layer to 120 cm depth. Coarse root biomass for *A. sphaerocephala* was negatively correlated with soil moisture (*p* < .01), but that for *A. intramongolica* was positively related (*p* < .01, Figure 2b). Coarse root biomass for *A. sphaerocephala* was not significantly correlated with total soil nitrogen content (*p* > .05), while the correlation for *A. intramongolica* was positive (*p* < .01, Figure 2c). Coarse root biomass for both species was positively correlated with total soil carbon content (*p* < .01, Figure 2d).

**Figure 3:**
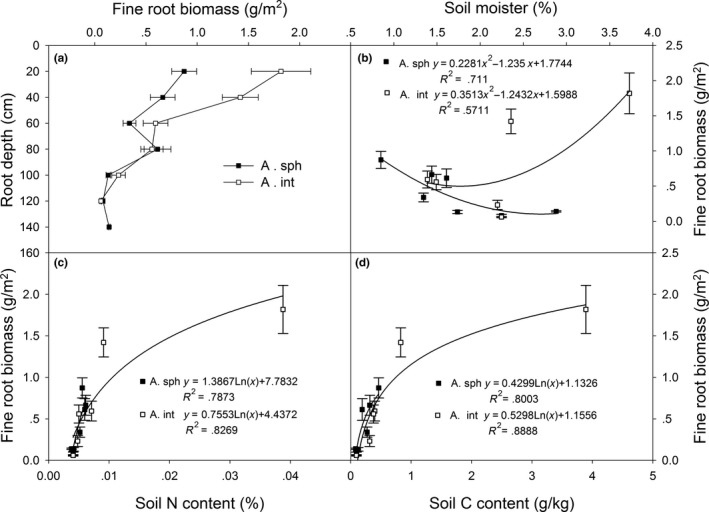
The fine root biomasses for *Artemisia sphaerocephala* (A. sph) and *A. intramongolica* (A. int) (a) and their relationships with soil moisture (b), total soil nitrogen (c), and total carbon content (d) in the Hunshandake desert, China

Unlike coarse roots, fine root biomass for the two *Artemisia* species varied in similar patterns (Figure 3a), with more fine root biomass for *A. intramongolica* in the first two layers. Fine root biomass of *A. intramongolica* in the first two layers was 69% of the total fine root biomass, while that of *A. sphaerocephala* was 54%. Both species had relatively greater fine root biomass in 80 cm deep, which was about 22% of the total fine root biomass for *A*. *sphaerocephala*. Fine root biomass for the two species did not significantly correlated with soil moisture (*p* > .05, Figure 3b), but positively correlated with both total soil nitrogen content (*p* < .01, Figure 3c) and total soil carbon content (*p* < .01, Figure 3d), respectively.

### 3.4 Root biomass allocation

**Figure 4:**
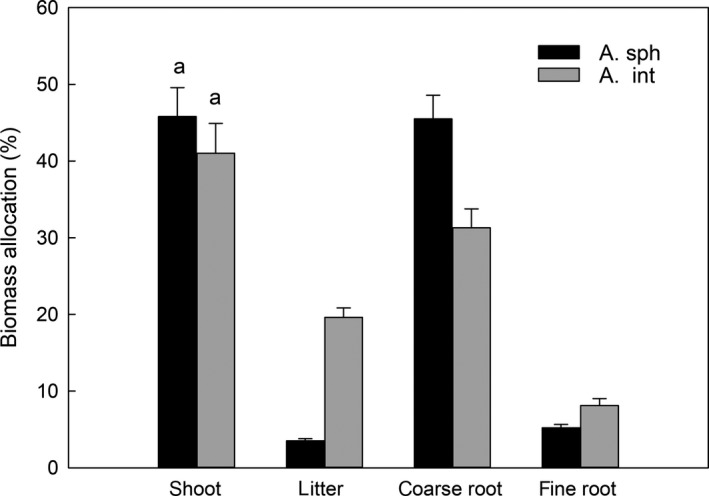
Component biomass allocation of *Artemisia sphaerocephala* (A. sph) and *A. intramongolica* (A. int) in the Hunshandake desert, China. Bars with similar letters indicate no significant differences between species (*p* > .05)

Average root biomass allocation was 51% and 39% for *A. sphaerocephala* and *A. intramongolica*, in comparison to total plant weight (Figure 4). Coarse root biomass allocation for *A. sphaerocephala* was as high as 46%, while that for *A. intramongolica* was only 31%. Fine root biomass allocation for the two species, however, was much lower at 5%–8%. The average shoot biomass allocation was 46% and 41% for *A. sphaerocephala* and *A. intramongolica* (*p* > .05), respectively. *A. intramongolica* had much higher litter biomass than *A. sphaerocephala*: 20% versus 4% of the total biomass. The values of root‐to‐shoot ratios were about 1.1 and 0.9 for *A. sphaerocephala* and *A. intramongolica*, respectively.

## 4 DISCUSSION

A number of studies have demonstrated that some plant species are able to send roots very deep in soil, especially the plants that grow well in summer drought or desert plants that grow for years with minimal or no rainfall (Canadell et al., 1996; Wang & Gao, 2003). Plant root depth and biomass are determined primarily by both abiotic factors, for example, soil moisture, soil texture, nutrient availability (Asseng, Ritchie, Smuchie, & Robertson, 1998; Peng, Guo, & Yang, 2017; Purbopuspito & Van Rees, 2002), and biotic factors, such as species, root life span, and root competition (Canadell et al., 1996; Casper & Jackson, 1997; Jackson, Manwaring, & Caldwell, 1990; Kobiela, Biondini, & Sedivec, 2016; Maina, Brown, & Gersani, 2002; Thoms, Köhler, Gessler, & Gleixner, 2017). In present study, because of desertification, drought and poor soil was intensified in the desert, with average surface soil moisture and nutrients from the *Artemisia* sample sites were only 10%–40% and 4%–18% of the nearby steppe, respectively. Relative great root depth (Figure 1a) and biomass allocation (Figure 4) for the two *Artemisia* species in the desertification land with low soil moisture and poor nutrients supported the hypothesis that plants preferentially allocate more resources to roots when water and nutrients become limited to growth (Garnier, 1991). *Artemisia sphaerocephala* and *A. intramongolica* are two typical semishrubs, experiencing similar light regimes and precipitation, but differing in soil conditions, for example, soil moisture, soil nitrogen, and carbon contents (Table 1). The root vertical distribution patterns for the two *Artemisia* species differed considerably with the heterogeneity of soil conditions in the desert. In this study, coarse root diameters, coarse and fine root biomass for *A. intramongolica* growing in relatively moist fixed dunes, dropped sharply from the surface to the IV layer, while those for *A. sphaerocephala* reduced gradually from the surface to VII layer (Figure 1a). Relatively higher surface soil moisture may enhance surface root growth for both species, while dry soil may cause roots to grow deeper. There is evidence of this phenomenon in this study in the positive relationships of coarse root diameters and coarse root biomass with soil moisture for *A. intramongolica* (Figures 1b and 2b). In mobile sandy dunes, soil moisture in surface layers was much low, mainly due to strong evaporation, low capacity of water content (Table 1), and low canopy cover. These resulted in the strongly and negatively relations of coarse root diameters and coarse root biomass with soil moisture for xeric *A. sphaerocephala* (Figures 1b and 2b). This may also explain the fact that *A. sphaerocephala* had greater coarse root diameter and biomass in the deep layers.

The root depths for both *Artemisia* species were relatively shorter than those reported by Nakamura et al. (2002) and Richards and Caldwell (1987), and this may be primarily due to the high water table, which is about 1.5 m or less in the region. Lewis and Burgy (1964) showed that the survivorships of some species in arid systems depend completely on a plant's ability to tap water from permanent water tables. High water table in the region resulted in relatively greater fine root biomass allocation in deep layer (>80 cm) for both species, as well as no significant correlations (*p* > .05, Figure 3b) between fine root biomass and soil moisture changes with depth. Higher root biomass or root biomass allocation for plants in arid systems can enhance the capacity of water uptake from deep soil and root water storage (Wang & Gao, 2003). Large investment in roots may also increase carbohydrate storage capacity, potentially improving recovery after drought. However, high root biomass allocation may reduce primary production because of increasing maintenance respiration by living cells contained in roots (Callaway, Delucia, & Schlesinger, 1994; Wang & Gao, 2003). Further studies on the other traits for these species such as tissue morphology, transport and absorptive fine root ratio and root water storage are needed.

Plant root is a central component of global nutrient cycles, and root development is remarkably sensitive to the variations of inorganic nutrient supply and distribution in soil (Forde & Lorenzo, 2001; Friend, Eide, & Hinckley, 1990; Jobbagy & Jackson, 2001). Both total soil N and C contents in surface layers (0–40 cm) for the mobile dunes were about 4% of the adjacent *stipa* steppe, while those for the fixed dunes were relatively higher, about 16% and 24% (Table 1). Poor soil nutrients, especially soil N and C (Wang & Gao, 2003), are often critical factors for plant root development in a desert region. In this study, root diameter and root biomass were positively correlated with total soil nitrogen and carbon contents for the two species in the desert (Figures 1c,d, 2c,d, 3c,d). These results suggest that poor soil nutrients, combined with soil moisture, may be the limiting resources for survival and settlement for the two species. This may provide reasonable basis for species selection in desertification land restoration in the Hunshandake desert, for example planting *A. sphaerocephala* on the dry mobile dunes and *A. intramongolica* on the low moist sand lands. Because of the confounding factors of soil moisture, soil nutrients, and species differentiation, further studies, for example, reciprocal transplanting, manipulation of water, fertility and greenhouse cultivation, are needed to explore the fundamental bases of plant differences in root traits and their relations with environmental changes in the Hunshandake desert.

## 5 CONCLUSIONS

The significant divergences in the root traits, for example, coarse root distribution patterns, root biomass in the first two layers, and their relationships with soil moisture, revealed their diverse in growth patterns and adaptation strategies to considerable heterogeneity of soil conditions in the desert. Relative deep root, high root‐to‐shoot ratio, and significant negative relationships of coarse root biomass with soil moisture for *A. sphaerocephala* exhibited its higher capacity in drought tolerance than *A. intramongolica*, and more suitable for desertification lands recovery and reconstruction. In general, the positively relations of root traits with total soil nitrogen and carbon contents for the two *Artemisia* species suggest that both soil moisture and poor soil nutrients were the limiting resources for plant growth and survival in the Hunshandake desert. Therefore, both the soil moisture and nutrients should be seriously considered in the process of sandy land restoration with the two *Artemisia* species in dryland ecosystems in north China.

## CONFLICTS OF INTEREST

None declared.

## AUTHOR CONTRIBUTION

Renzhong Wang conceived and designed the experiments. Linna Ma wrote the main manuscript text and analyzed the data. Xiaoqiang Liu and Xiuli Gao performed the experiments and processed the data. All authors reviewed the manuscript.

## Data Availability

All data are included in the manuscript. The data are uploaded in Dryad, Dataset, https://doi.org/10.5061/dryad.9zw3r22b4
